# Bridging the language divide in health

**DOI:** 10.2471/BLT.15.020615

**Published:** 2015-06-01

## Abstract

While the English language dominates much of the world’s public health information, several initiatives are underway to provide such information in other widely spoken languages. Patrick Adams and Fiona Fleck report.

“A close relative had been diagnosed with a rare disease. We searched for information on it in Arabic and found websites that were unstructured or were essentially chat forums,” recalls Dr Majid Altuwaijri.

“But when we searched in English we found a wealth of good quality information.”

As co-founder of the Saudi Association for Health Informatics, Altuwaijri was well placed to help his relative, given his expertise in information technology and fluency in English.

However, globally only an estimated 600 to 700 million people have English as a second language, like Altuwaijri, in addition to some 335 million native English speakers, with varying degrees of fluency.

That leaves most of the world’s population – some six billion people – with little or no access to a large body of public health information because it is in English.

Language can be a barrier to accessing relevant and high quality health information and delivering appropriate health care – an unmet need that is amplified on a global scale.

**Figure:**
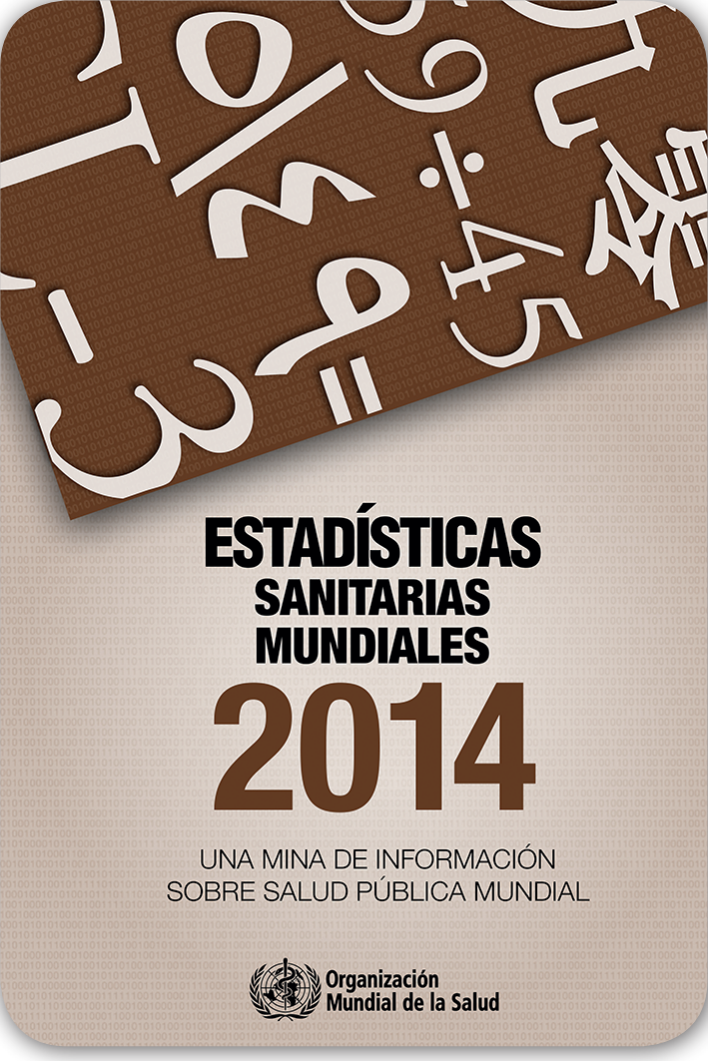
**Cover of the WHO publication the World Health Statistics 2015 – one of the WHO publications that is published in all six official United Nations languages.**

Source: WHO.

“The trend towards monolingualism is far from decreasing, with the hegemonic use of one language, English, over the other five United Nations (UN) languages,” the UN Joint Inspection Unit concluded in a 2011 report on implementation of multilingualism in UN organizations.

“The trend towards monolingualism is far from decreasing, with the hegemonic use of one language, English, over the other five United Nations languages.”

UN Joint Inspection Unit

As part of the UN system, the World Health Organization’s (WHO) six official languages – Arabic (242 million native speakers), Chinese (1197 million), English (335 million), French (76 million), Russian (16 million), and Spanish (399 million) – are the first languages of only 2.4 billion people, according to *Ethnologue: Languages of the World,* 18th edition – less than half the world’s population.

In addition, German (78 million native speakers) is an official language in WHO’s European Region and Portuguese (203 million) in WHO’s African, European and Americas Regions. For native speakers of other languages, such as Hindi (260 million native speakers) and Bengali (198 million), the unmet need for health information may be great.

English has long been the lingua franca of scientists – including those working in public health – and while more WHO publications and web pages are produced in English than in any other language, WHO publications appear in more than 70 languages.

All WHO’s official documents, such as World Health Assembly reports and resolutions, are translated into the six official languages, but this is not the case for the rest of WHO’s publishing output, including technical reports and clinical guidelines. Moreover, WHO launched its six-language multilingual website in 2005, but most of its web content is still in English.

While Portuguese is the world’s sixth most spoken language (after Chinese, English, Hindi, Spanish and Arabic), most Portuguese-speaking scientists seek to publish their work in English to gain wider circulation, according to a study published in a report for the European Molecular Biology Organization in 2007 by Rogerio Meneghini.

In public health, the linguistic disconnect between those providing health information and those who need that information affects everyone from clinicians and patients to public health managers and policy-makers.

One of the most popular health information websites, Wikipedia, collaborates with Translators Without Borders to bridge that divide. With the help of the global network of translators, Wikipedia Medicine has built a large collection of articles in more than 100 languages and has at least some medical content in more than 250 languages.

“We did a lot of work for the Ebola outbreak with Translators without Borders and others because most information on Ebola was in English, which is only spoken by 15–20% of the population in West Africa,” says Wikipedia editor Dr James Heilman, adding: “Now we have content on Ebola in around 115 languages.”

In addition, some United States-based websites provide multilingual health information, such as the Health Information Translations collaboration and the National Network of Libraries of Medicine.

While a few projects are making more multilingual public health information available in many widely spoken languages, the overall dearth of such information produces a divide between the health-information haves and have-nots that is exacerbated by poor internet connectivity and unreliable electrical supplies in developing countries.

For Dr Alfredo José, director of the national library in Mozambique, health professionals in this Portuguese-speaking country – where half the population lives below the poverty line – have often felt linguistically isolated, surrounded by Anglophone countries.

In 2005, WHO established the ePORTUGUESe programme, to increase access to health information in Portuguese as part of a collaboration with Angola, Brazil, Cabo Verde, Guinea Bissau, Mozambique, Portugal, Sao Tome & Principe and Timor-Leste.

“We started by developing a national virtual health library in each of the eight Portuguese-speaking countries, which can be adapted to local needs and conditions,” says Dr Regina Ungerer, who has led the programme for the past 10 years.

“The virtual library allows countries to have their own technical and scientific portal providing a local directory of health events, websites and legislation that can be accessed by anyone with an internet connection,” Ungerer says.

The virtual library platform was developed by the Latin American and Caribbean Center on Health Sciences Information best known by its acronym as BIREME, part of WHO’s regional office for the Americas.

“With the ePORTUGUESe network, we are empowered,” says José. “Brazil and Portugal contribute documents to the virtual health library, and we’re connected to other Portuguese-speaking countries through the online discussion group.”

“With the ePORTUGUESe network, we are empowered.”

Dr Alfredo José

A network of documentation centres globally provide WHO publications in local languages, including 30 centres across the European region alone; WHO works closely with governments and collaborating centres on an ad hoc basis to translate its publications into local languages.

The documentation centre in Moscow, established in 1994, has played a key role in providing WHO publications in the Russian language online and in print, as part of a collaboration with the Russian government.

**Figure:**
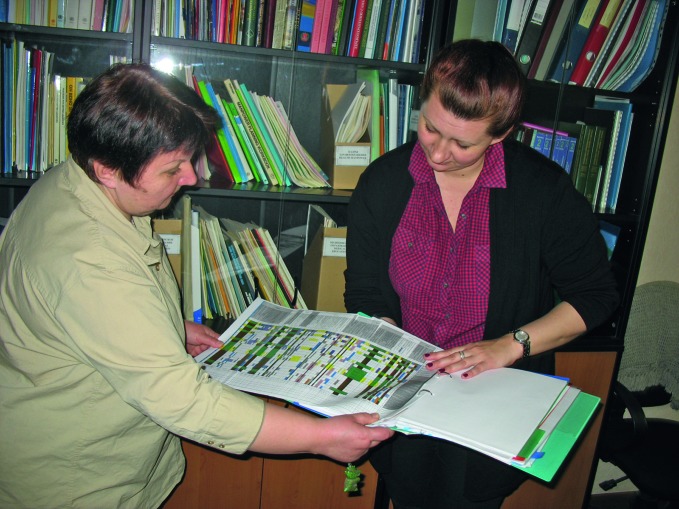
**Tatyana Kaigorodova (left) and Katerina Zimina working in the WHO documentation centre archive in Moscow.**

Source: WHO.

A recent analysis showed that 70% of the 880 251 people, who used the centre’s online library between October 2009 and May 2014, were from the Russian Federation, 10% from Ukraine, 6% from Kazakhstan and 3% from Belarus, while users from other countries constituted less than 1% per country.

“We were amazed to see we had users from 50 countries, not only from the Commonwealth of Independent States, but also Latvia, Israel, the United States, the United Kingdom and other countries,” says Tatyana Kaygorodova, who runs the WHO Moscow documentation centre.

In 2012, WHO established a programme funded by the Russian government to increase the number of technical WHO publications in Russian, such as clinical guidelines, and to establish a mechanism for consulting Russian-speaking public health experts on which publications they needed most.

An important part of the project is to improve the quality of translation to reduce the risk of clinical errors by inviting Russian experts in the relevant fields to review the clinical content of the translations before they are published, Kaygorodova says.

“If it's a translation of a press release, for example, then approximation is fine. But translations of guidelines for health professionals must be precise and accurate because mistakes can kill,” she says.

What these WHO programmes have done for Portuguese and Russian-speaking countries and people, WHO’s Global Arabic Programme has sought to do for health professionals throughout the Arabic-speaking world.

The WHO Arabic Programme aims to disseminate the work of WHO through Arabic publications, make reliable and current health information and research outcomes available in Arabic, and establish networks and knowledge communities in Arabic translation, terminology and publishing.

Although Arabic is an official UN language, Arabic speakers still struggle to access reliable health information in their native language. Searching in vain for reliable health information in Arabic “was what triggered the idea for our study,” says Altuwaijri.

The study published in 2009 by Altuwaijri and colleagues at King Saud bin Abdulaziz University for Health Sciences in Saudi Arabia, in collaboration with the Health on the Net Foundation and the University of Geneva, found that just over 4% of all Arabic health information websites met international quality standards. 

In light of their findings, Altuwaijri and colleagues recommended the establishment of an Arabic health information foundation to govern and accredit Arabic health websites and an Arabic health encyclopedia.

Initiated in 2010 by King Saud bin Abdulaziz University and the Saudi Association for Health Informatics, the *King Abdullah bin Abdulaziz Arabic Health Encyclopedia* came together with support from several international partners. 

Comprised of translations contributed by health professionals from across the Arab world, the encyclopedia is still in its early stages, but Altuwaijri believes it’s already having an impact: “The doctors I’ve spoken with, once they’ve been introduced to the encyclopedia, love it.”

