# Corrigendum

**DOI:** 10.2471/BLT.15.100715

**Published:** 2015-07-01

In Volume 93, Issue 6, June 2015, page 365, the eighth paragraph should read: “… Russian (161 million) …”.

